# Editorial: The prevention of HIV among adolescent girls and young women: leaving no one behind

**DOI:** 10.3389/fpubh.2026.2005756

**Published:** 2026-10-27

**Authors:** Enos Moyo, Tafadzwa Dzinamarira

**Affiliations:** 1 Africa Centre for Inclusive Health Management, Faculty of Economic and Management Sciences, Stellenbosch University, Stellenbosch, South Africa; 2 School of Health Systems and Public Health, Faculty of Health Sciences, University of Pretoria, Pretoria, South Africa

**Keywords:** adolescent girls and young women, criminalization, HIV prevention, HIV self-testing, pre-exposure prophylaxis, sexuality education, sub-Saharan Africa

In 2024, about 4,000 adolescent girls and young women (AGYW) aged 15 to 24 years acquired HIV each week, and roughly 3,300 of those infections occurred in sub-Saharan Africa (1). We proposed this Research Topic because that figure has not kept pace with the tools now available. Oral PrEP has been recommended for nearly a decade, HIV self-testing is widely approved, and WHO added twice-yearly injectable lenacapavir to its prevention guidance in 2025 (2). We invited work that asked who is still being missed, and why. The nine articles cover eight countries and include one global analysis. Together, they suggest the difficulty lies less with the products than with the gap between a method being available and a young woman being able to continue using it for as long as she needs to.

In their opinion article, Murewanhema et al. argue that gender inequality, poverty, restrictive social norms, and a shortage of youth-responsive services continue to drive risk, and that current efforts fall short of the scale of the problem. Lian et al. provide the epidemiological backdrop. Drawing on Global Burden of Disease 2021 data for HIV, syphilis, and hepatitis B among women of childbearing age and children under five, they find the heaviest burdens in regions with lower sociodemographic development. Although the study covers a broader population, it links AGYW prevention to maternal health and vertical transmission. It shows how national averages can overshadow the populations this Research Topic aims to identify.

Two studies examine what young people know and how able they feel to act on that knowledge. Barchi et al. surveyed school-going adolescent girls in northwestern Botswana, most of whom had not had sex. Some were unsure about correct condom use or held misconceptions about HIV. Girls who felt confident discussing condoms and refusing condomless sex were more likely to believe they could insist on condom use, which argues for sexuality education that begins before sexual debut and gives girls room to practice these conversations. Among sexually experienced students in Fuzhou, China, Yang et al. found that stronger family functioning was associated with more favorable attitudes toward HIV testing and more information-seeking, with self-monitoring explaining part of that association. The sample was not designed around young women, but the findings bear on how caregivers might support daughters. Family-based approaches should protect young people's privacy and recognize that not every home is safe.

The widest gap these studies expose lies between willingness and sustained use. In an informal settlement in Kampala, Segawa et al. found that young people rated HIV self-testing highly, yet far fewer had used a kit; cost, patchy availability, doubts about the result, and the opinions of peers and family all stood in the way. Moyo et al. report that 81.1% of eligible participants in Namibia's DREAMS program started oral PrEP. What struck us was who did not. Young women with recent sexually transmitted infections, multiple or concurrent partners, or partners of unknown HIV status were less likely to initiate, as were those in some smaller towns. The pattern calls for locally designed counseling and service delivery, not national messaging alone.

Heffron et al. evaluated PrEP delivered through postabortion care in Kenya, where most clients were in their twenties. Just over half received any PrEP information, and persistence among those who started was low. Clinics randomized to make scheduled phone calls retained significantly more women at 1 month, though the difference in refills, the trial's primary outcome, was not statistically significant. For anyone planning integration, the harder work begins after the first visit.

Choice and safety are the concern of the last two empirical studies. Kerrigan et al. found low awareness and use of PrEP among cisgender women in the Dominican Republic and the United States. Many participants nonetheless preferred a long-acting injectable to daily tablets, with preferences shaped by concerns about injections, stigma, partner violence, and prior use of injectable contraception. The population is broader than AGYW, but with lenacapavir now recommended, programs for younger women will soon face the same questions. Wamoyi et al. showed how criminalization narrows choice. Young female sex workers in Tanzania described stigma, mistreatment, and fear of arrest, and leaned on peers to decide where, and whether, to seek care. For them, a better-equipped clinic offers little if attending it carries legal risk.

Figure 1 outlines the pathway that links these findings.

**Figure 1:**
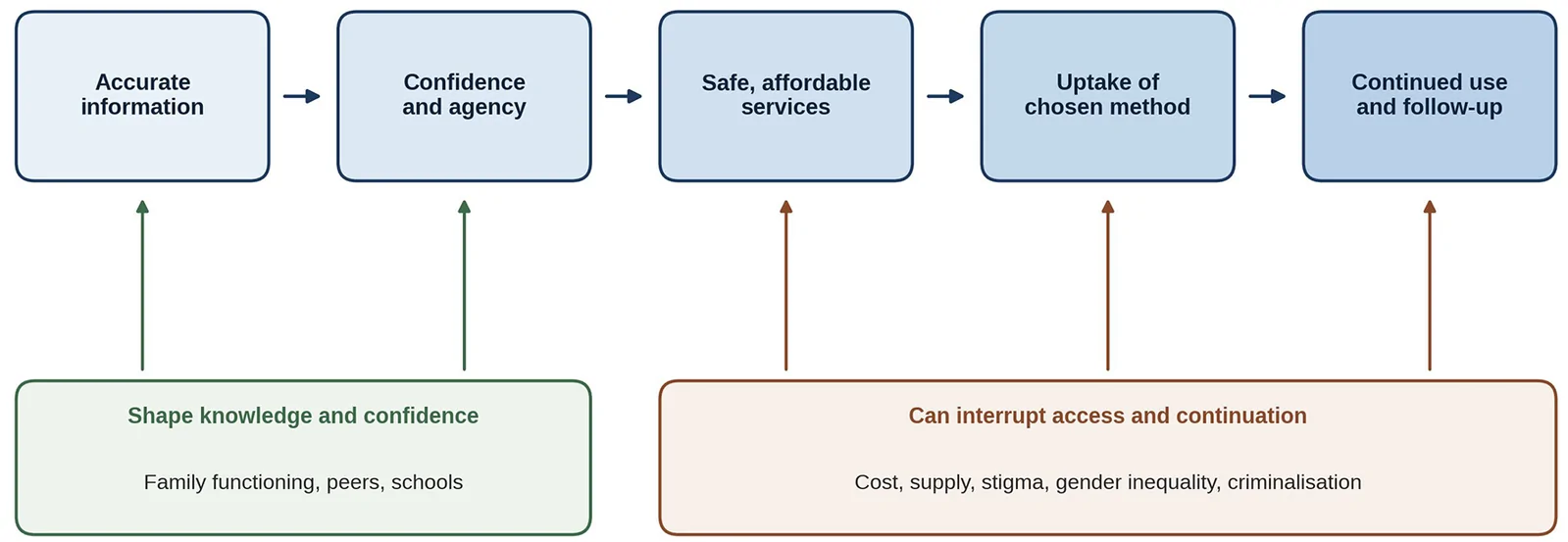
Conditions for effective HIV prevention among AGYW. Each stage depends on the one before it. Family functioning, peers, and schools shape knowledge and confidence, while cost, unreliable supply, stigma, gender inequality, and criminalization can interrupt access, uptake, and continued use.

Initiation figures say little on their own. Monitoring should follow young women beyond the first visit and report results by age, place, and circumstance, so that groups like those identified by Moyo et al. and Wamoyi et al. are counted rather than averaged away. The Research Topic also has gaps: none of the articles centers on male partners, on girls younger than 15, on economic support, or on delivering injectable PrEP in African settings. These gaps have become more pressing since reductions in international funding in 2025 closed or suspended many prevention services built for young women (3).

We thank the authors, reviewers, and the Frontiers editorial office. We hope readers will find in these articles a practical account of where prevention for AGYW breaks down and what it would take to strengthen it.
